# The Leeds General Infirmary

**Published:** 1918-12-07

**Authors:** Charles Lupton

**Affiliations:** Chairman and Treasurer of the General Infirmary at Leeds. 34 Albion Street, Leeds


*To the Editor of* THE HOSPITAL.

### The Leeds General Infirmary.

*To the Editor of* THE HOSPITAL.

SIR,—Whilst thanking you for your sympathetic notice of the report, which has recently been adopted, on the nursing conditions at the General Infirmary at Leeds, I should like to enter a mild protest against your concluding statements. From these it might be inferred that the Leeds Infirmary has hitherto not been a really well-managed modern training-school and that it does not possess a good nurses' home.

On the first point I should ask that our tree may be judged by its fruits; outside testimony received by me from many quarters previously to the war and many reports which I have received during the war from persons unconnected with our training-school convince me that the nurses turned out from it compare, to say the least, favourably with the nurses trained in other great training-schools; whilst so far as the second point is concerned we already possess a nurses' home, the last portion of which was completed only two years ago and has been in regular occupation since, which for comfort and completeness will compare favourably with any other nurses' home that I know.

It is quite true that our lady superintendent has been for some time past dissatisfied with our present nurses' dining-hall, and that she has also been anxious to improve the conditions under which the nurses have worked in other respects. Her continuous aim has been to increase their opportunities for self-improvement and innocent recreation and to raise the general status of the nursing profession, but some of her recommendations have only been rendered necessary by the increased responsibility thrown upon nurses owing to the war, and others have been long since determined on and would have been carried into effect but for the war. I feel sure that you will agree that it would be unfortunate if a rather bold effort to bring a great training-school up to the highest pitch of excellence in the middle of a period of the greatest financial difficulty should be construed into an admission of past mismanagement on the part of those who had the previous control of the institution.—Yours truly,

CHARLES LUPTON,
Chairman and Treasurer of the General Infirmary at Leeds.

34 Albion Street, Leeds, December 3.

[We have great pleasure in publishing Mr. Charles Lupton's letter, which is an interesting account of the actual condition of the Leeds General Infirmary training-school and its nurses' homes. We have always taken the greatest interest in this Leeds school, and have followed its progressive development to its present state of expansion and betterment with continuous satisfaction. Mr. Charles Lupton is an ideal chairman, who has devoted much of his time and all his talents to everything calculated to promote the best interests of the General Infirmary and all associated in its work. We are not in sympathy with one direction in which the emoluments of probationers at the moment are being largely increased by some hospital schools, whilst the payment of fully-trained nurses and sisters is, in too many cases, at any rate, left at a much lower scale than it ought to be, having regard to the nature and responsibilities of their work. Experience teaches that candidates for the profession of trained nurses should, like pupils in other professions, be glad to give such services as they can render in the early days of their training with a mere pocket-money honorarium. On the other hand, speaking generally, the emoluments, hours off duty, and holiday allowances of the sisters and fully-trained nurses should be raised to a

**The Editor's Letter-Box**—(*continued*).

more adequate standard. The invaluable work and services rendered by conscientious and devoted women who have passed through their training-school and hospital training with marked success and obtained their certificates as fully-trained members of the nursing profession, entitles them to greater remuneration.—Ed. The Hospital.]

